# A Thermodynamic Study on the Interaction between RH-23 Peptide and DMPC-Based Biomembrane Models

**DOI:** 10.3390/membranes12121282

**Published:** 2022-12-19

**Authors:** Cristiano Giordani, Stefano Russo, Cristina Torrisi, Silvia Morante, Francesco Castelli, Maria Grazia Sarpietro

**Affiliations:** 1 Grupo Productos Naturales Marinos, Facultad de Ciencias Farmacéuticas y Alimentarias, Universidad de Antioquia, Calle 70 No. 52-21, Medellín 050010, Colombia; 2 Instituto de Física, Universidad de Antioquia, Calle 70 No. 52-21, Medellín 050010, Colombia; 3 Dipartimento di Scienze del Farmaco e della Salute, Università degli Studi di Catania, Viale A. Doria 6, 95125 Catania, Italy; 4 Dipartimento di Fisica, University of Rome Tor Vergata and Istituto Nazionale di Fisica Nucleare (INFN), Via della Ricerca Scientifica 1, 00133 Roma, Italy

**Keywords:** RH-23, anticancer peptide, DMPC, MLVs, langmuir minitrough, differential scanning calorimetry

## Abstract

Investigation of the interaction between drugs and biomembrane models, as a strategy to study and eventually improve drug/substrate interactions, is a crucial factor in preliminary screening. Synthesized peptides represent a source of potential anticancer and theragnostic drugs. In this study, we investigated the interaction of a novel synthesized peptide, called RH-23, with a simplified dimyristoylphosphatidylcholine (DMPC) model of the cellular membrane. The interaction of RH-23 with DMPC, organized either in multilamellar vesicles (MLVs) and in Langmuir-Blodgett (LB) monolayers, was assessed using thermodynamic techniques, namely differential scanning calorimetry (DSC) and LB. The calorimetric evaluations showed that RH-23 inserted into MLVs, causing a stabilization of the phospholipid gel phase that increased with the molar fraction of RH-23. Interplay with LB monolayers revealed that RH-23 interacted with DMPC molecules. This work represents the first experimental thermodynamic study on the interaction between RH-23 and a simplified model of the lipid membrane, thus providing a basis for further evaluations of the effect of RH-23 on biological membranes and its therapeutic/diagnostic potential.

## 1. Introduction

Cancer is one of the leading causes of death in the world [1]. Conventional, available therapies are mainly based on the destruction of tumoral aggregates, but lack of specificity is responsible for numerous side effects [2,3]. The presence of peptide-specific receptors in the membrane of cancer cells, as well as the discovery of peptides that influence the development of tumors, have led to the discovery of new non-conventional anticancer drugs with high selectivity and efficacy [4]. These peptides can be employed not only as therapeutic molecules, but also as carrier systems for cytotoxic drugs [5]. Cancer cells express antigens that lead to the activation of the immune system, which has triggered the development of peptide-based vaccines as novel anticancer agents. These vaccines, homologues of said antigens, stimulate the production of T cells (T helper or T-killer cells) that interact with tumor antigens [6,7]. It is then possible to monitor the effectiveness of vaccination by determining antigen-specific T cell responses. However, one of the major disadvantages of peptide-based vaccines is weak immunogenicity due to mutations of the original antigen [8]. Peptides that show anticancer activity in animals are found mainly in the immune system and in the central nervous system, as well as in the digestive system, heart, bones, muscles, and skin. Said peptides express their action through a number of different ways, such as inhibition of tumor angiogenesis [9], induction of tumor apoptosis [10,11], induction of cell necrosis [12], and immunomodulatory function [2].

Antimicrobial peptides (AMPs) are cationic and amphiphilic peptides with cytotoxic properties [13,14,15]. In mammals, AMPs often show weak antimicrobial activity under physiological conditions, but they can modulate the immune system response through various mechanisms. Defensins and cathelicidins are examples of mammalian endogenous AMP families that contribute to cell defense by destroying bacterial cell membranes [16,17]. In particular, cathelicidins act as zymogens that, after proteolysis, release a variety of active AMPs. The only known type of human cathelicidin is hCAP-18, the C-terminal domain of which is cleaved upon extracellular lysis by proteinase 3 (PRTN3) to produce the AMP known as LL-37 [18,19]. Although LL-37 shows a broad spectrum of antibacterial properties, it displays an important limitation in clinical applications because of its high affinity for the internal walls of blood vessels [20]. The binding process is favored by the presence of heparan sulfate, an anionic polysaccharide that resides in the outermost membrane of the endothelium and in the extracellular matrix. On the other hand, numerous studies have reported a certain cytotoxic activity against tumoral cells by peptides that attach to heparan sulfate [21,22].

The LL-37 peptide at physiological pH 7.4 is characterized by a net positive charge equal to +6. In these conditions, its secondary structure is mainly in an alpha-helix configuration [23]. Due to the alternation between positive and hydrophobic amino acids, LL-37, as previously mentioned, is able to bind to the membrane of blood cells by interacting with heparan sulfate molecules. This feature makes it unsuitable as an antitumoral candidate since it would get stuck before reaching target tumor cells; additionally, if loaded with a radionuclide, it would release radiation into the wrong tissues [24]. To overcome these problems, the amino acid sequences located in the N-terminal domain (responsible for binding heparan sulfate) and in the random-coil portion of the C-terminal domain, namely the first 6 and last 8 amino acids of the peptide chain, can be eliminated. Subsequently, a 23-amino acid shorter peptide with secondary alpha-helix structure can be obtained, held in place by the replacement of all the glycine residues with alanine residues [25]. Furthermore, to increase the selectivity of the peptide towards tumoral membranes, the positive amino acids K2, K6, K12, R17, K19, and R23 can be replaced with histidine residues; by doing so, the secondary nitrogen atom of the imidazole ring is protonated at acidic pH. The resulting peptide will therefore be almost neutral at physiological pH, e.g., in near healthy cells, and positively charged when located in proximity of the acidic extracellular environment usually generated by cancer cells. However, its neutrality at physiological pH makes it less soluble. To overcome this problem, two phenylalanine residues can be replaced with two more hydrophilic, equivalent tyrosine residues, thus obtaining the final peptide named RH-23, whose primary sequence is RHSKEHIAKEYHRIVQHIHDYLH [26].

In order to shed light on the mechanisms of action between peptides and eukaryotic cell membranes, it is very helpful to use a simplified model of cellular membranes since the complexity of the eukaryotic membrane structure is seriously difficult to replicate. This facilitates understanding the contribution of each singular component and evaluating the biological and physicochemical aspects of the interactions [27]. A simplified model of a cellular membrane for preliminary screening purposes can be prepared using a phospholipid such as DMPC (1,2-dimyristoyl-sn-glycero-3-phosphocholine), because PCs are highly abundant in mammalian membranes [28]. DMPC is one of the commonly used mammalian membrane mimetics due to its transition temperature, which is lower, but not too much lower, than the human internal temperature, which allows for optimal fluidity of the layers [29,30,31,32,33]. Even though a whole DMPC membrane model may be crude, it is an ideal candidate for preliminary studies on interplays between cellular membranes and peptides because of its easy preparation, reproducibility, and interpretation of results. The thermotropic properties of DMPC model membranes can be experimentally investigated using differential scanning calorimetry (DSC) on multi lamellar vesicles (MLVs) composed of DMPC, along with RH-23 inserted at different concentrations. Calorimetric results can give us indication of the thermotropism behind DMPC and RH-23 peptide interactions by analyzing variations in DMPC transition temperature and enthalpy, both as a function of the molar fraction of the RH-23 peptide [34,35]. The Langmuir-Blodgett (LB) technique can be employed to form DMPC monolayers at the air/water interface which, by measuring variations in surface pressure in relation to the mean molecular area (at different peptide concentrations), enables the evaluation of the miscibility/immiscibility of RH-23 peptide with DMPC monolayers [36,37,38,39].

In our experiments, we employed a combined DSC/LB approach in order to study the interaction between RH-23 peptide and DMPC biomembranes at physiological pH. This work represents the first experimental thermodynamic study of the interaction between RH-23 and a simplified model of a lipid membrane. Our preliminary data clarify the mechanism of action of the peptide, showing that RH-23 was distributed among MLV bilayers and influenced the cooperation between DMPC monolayers.

## 2. Materials and Methods

### 2.1. Materials

RH-23 peptide (purity > 95%) was supplied by Giotto Biotech S.r.l. (Sesto Fiorentino, Italy). 1,2-Dimyristoyl-sn-glycero-3-phosphocholine (DMPC) (purity 99%) was supplied by Genzyme Pharmaceuticals (Liestal, Switzerland). Chloroform and tris-hydroximethyl-aminomethane (TRIS) were obtained from Merck (Darmstadt, Germany). A KSV Langmuir minitrough film balance apparatus (KSV Instruments Ltd., Espoo, Finland) was used. This apparatus includes a trough (24,225 mm^2^ available area for monolayer formation) coated with polytetrafluoroethylene (Teflon) and equipped with a water jacket to provide temperature control and two mechanically coupled barriers of hydrophilic Delrin.

### 2.2. MLV Preparation

MLVs containing different molar fractions of the RH-23 peptide were prepared as follows. DMPC was solubilized in chloroform/methanol (1:1, *v*/*v*). RH-23 peptide was solubilized in methanol. Aliquots of DMPC (containing 0.01032 mmol) were delivered in glass tubes. Aliquots of RH-23 peptide solution were added to the tubes glass containing DMPC. Aliquots of RH-23 peptide were added in order to obtain the following molar fractions (in respect of DMPC) of the peptide for different glass tubes: 0.03, 0.045, 0.06, 0.09, and 0.12. Then, phospholipid/peptide films were prepared through evaporation of the solvents under nitrogen flow at 37 °C. The obtained mixed films were kept in a freeze dryer for at least two hours to eliminate the solvents. A 168 μL volume of a TRIS solution (5 mM, pH 7.4) was added to the film. Each sample was kept in a water bath at 37 °C for 1 min and vortexed for 1 min. The process was repeated three times. Finally, samples were left for 1 h at 37 °C. MLVs without RH-23 were also prepared, as a reference, following the same procedure described above [40,41,42].

### 2.3. Surface Pressure/Mean Molecular Area Isotherms

Film balance measurements were performed using the KSV minitrough apparatus, which includes a trough (24,225 mm^2^ available area for the monolayer formation) coated with polytetrafluoroethylene (Teflon), two mechanically mobile coupled hydrophilic barriers (coated in Delrin), a platinum surface pressure sensor, a computer interface unit, and operating software. This system was connected to a circulating water bath to keep the temperature constant at 37 °C (a temperature mimicking human body temperature). The film pressure at the air/water interface was measured using the Wilhelmy plate arrangement attached to a microbalance. A subphase consisting of 5 mM Tris (pH 7.4) solution in ultrapure Millipore water (resistivity 18.2 MΩ cm) was used. The surface purity of the subphase was verified by closing and opening the barriers and ensuring that the surface pressure readings were not more than ±0.1 mN/m.

Solutions of DMPC in chloroform and RH-23 in methanol were prepared. Aliquots of 30 μL of the DMPC solution were spread drop by drop onto the aqueous subphase using a Hamilton syringe. Before use, the Hamilton syringe was cleaned three times with chloroform and then rinsed with the examined solutions. After waiting 15 min for solvent evaporation, the films were compressed with the two mobile barriers at a rate of 10 mm/min and the surface pressures/mean molecular area isotherms were recorded.

To evaluate the interaction of the peptide RH-23 with the DMPC monolayer, appropriate volumes of RH-23 solution (to give 0.17, 0.28, and 0.44 molar fraction of RH-23 with respect to DMPC) were added to the subphase. After waiting 10 min, the films were compressed at a rate of 10 mm/min. Preliminary experiments were performed to ascertain that methanol alone did not affect the DMPC monolayer.

To evaluate the ability of RH-23 to interact with the DMPC monolayer over time, the same procedure described in the previous point was repeated, but after waiting 10 min, the films were compressed until reaching 10, 20, or 30 mN/m. After waiting for half an hour, at the respective pressures, the barriers were closed until the lipid film collapsed.

Experiments were performed at a constant subphase temperature equal to the physiological temperature of 37 °C. This temperature was above the DMPC phase transition temperature so that the membrane was in a disordered, permeable state. In order to verify the robustness of our results, each experiment was repeated several times (at least three) to test for reproducibility.

### 2.4. Calorimetric Analysis

The DSC analysis was performed using the STAR^e^ thermoanalytical system (Mettler Toledo, Greifensee, Switzerland) equipped with a DSC822 calorimetric cell and MettlerTA-STAR software (version 16.00) for data acquisition and analysis. The calorimeter was calibrated using Indium (purity = 99.95%). The reference was prepared with 120 μL of TRIS buffer solution (50 mM, pH = 7.4) in a DSC aluminum pan (160 μL of total volume) and hermetically sealed. A 120 μL volume of the MLV dispersion containing 5 mg of DMPC was added to the aluminum calorimetric pan (160 μL). The pan was sealed and submitted to the following analysis: a heating scan from 5 to 37 °C at a rate of 2 °C min^−1^, followed by a cooling scan from 37 to 5 °C at a rate of 4 °C min^−1^. The heating and cooling scans were repeated not less than three times.

#### DSC Contact Kinetics

Contact kinetics were performed to verify the compound’s ability to interact with the DMPC biomembrane models. To carry out these measurements, an amount of RH-23 corresponding to the molar fraction of 0.12 was used. The solid compound was placed on the bottom of a 160 μL aluminum pan and 120 μL of aqueous suspension of MLVs was then added. The pans were hermetically closed and subjected to the following thermodynamic steps: (1) A calorimetric scan in heating from 5 to 37 °C with a rate of temperature increase of 2 °C/min; (2) An isothermal incubation period of 60 min at 37 °C; (3) A cooling phase from an incubation temperature of 37 °C down to a temperature of 5 °C at a rate of 4 °C/min. The whole procedure was repeated 9 times to evaluate the interaction of the compound with the MLVs over time.

## 3. Results and Discussion

### 3.1. DSC Measurements

**Figure 1:**
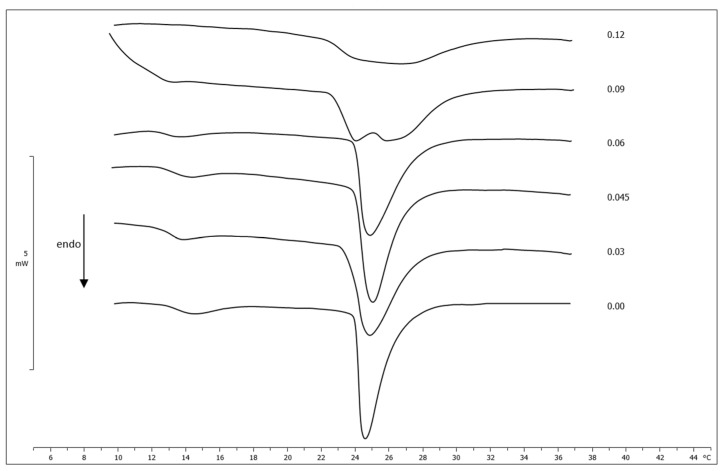
Calorimetric curves, in heating mode, of DMPC MLVs prepared with increasing molar fractions of the RH-23 peptide.

The interaction between the RH-23 peptide and DMPC MLVs was studied using differential scanning calorimetry. The DSC curves of the DMPC MLVs prepared in the presence of different peptide molar fractions were compared to the DSC curve of pure DMPC MLVs. This is shown in Figure 1. The DMPC MLV calorimetric curve showed a shallow pre-transition inflection at a temperature of approximately 14.4 °C due to the transition from the gel phase (ordered) to the ripple phase, and a pronounced transition peak at a temperature of 24.5 °C due to the transition from the ripple phase to the liquid crystal phase (disordered). Variations in these features with the peptide molar fraction indicated that RH-23 interacted with the DMPC MLVs.

The curves showed that the pre-transition inflection was visible up to a molar fraction of 0.06, tended to disappear at a value of approximately 0.09, and had completely disappeared from 0.12 upward. The transition peak moved towards slightly higher temperatures and broadened with the increase in the molar fraction of RH-23. Starting from a molar fraction of 0.09, a second peak at a lower temperature was visible. This indicated a phase separation with a non-homogeneous distribution of RH-23 in the MLV phospholipid bilayers. The enthalpy variation did not undergo significant changes, except at the molar fraction of 0.12, probably due to the effect of the high concentration of RH-23 on phospholipid cooperativity (Table 1). The shift of the main transition peak towards higher temperatures reflected the thermotropic behavior of MLVs when a substance interacted with the center of the phospholipid vesicle, which seemed to be stabilized by RH-23. This event often occurs when the lipophilic chains of an active compound intertwine with the acylic chains of phospholipids [43]. The decrease in enthalpy variation at a 0.12 molar fraction could be a result of disruption of van der Waals interactions, strengthening the hypothesis that insertion of the peptide, or at least a part of it, occurred in the hydrophobic core of the MLV [44].

We can conclude that the RH-23 peptide fit into the MLV phospholipid bilayers and interacted with them, thus stabilizing the gel phase, as highlighted by the increase in the temperature of the main peak.

**Figure 2:**
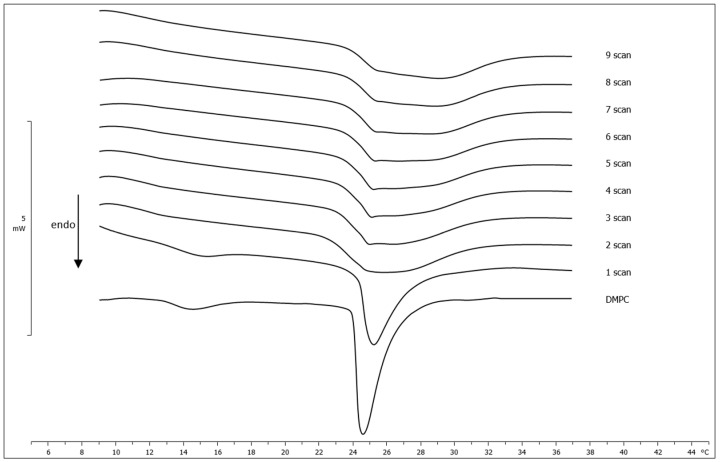
Calorimetric curves, in heating mode, of DMPC MLVs left in contact with RH-23 at a molar fraction of 0.12 at different incubation times.

The DSC technique was also used to study the ability of the RH-23 peptide to solubilize in the surrounding aqueous medium and, subsequently, reach and cross the lipid bilayers of the DMPC MLVs. Compound found in the aqueous phase, depending on its physicochemical characteristics, can be exposed to the surface of the MLVs and, subsequently, be incorporated into the lipid bilayer [45]. Any changes in the thermotropic parameters can therefore be related to the compound interacting with the lipid bilayers. If RH-23 was uniformly and completely dispersed in the phospholipid bilayer, the calorimetric curve would be similar to that obtained when the MLVs were prepared in the presence of the compound at a 0.12 molar fraction. Figure 2 shows the calorimetric curves of the MLVs of pure DMPC left in contact, at different incubation times, with the RH-23 peptide (at a molar fraction of 0.12).

The pre-transition peak disappeared after two hours of incubation while the main peak gradually moved towards higher temperatures and widened. Interestingly enough, the first scan closely resembled to the scan obtained from DMPC MLVs prepared with a 0.06 molar fraction of RH-23, while the final scan was clearly superimposable with the scan of DMPC MLVs prepared with a 0.12 molar fraction of the peptide. These results indicated that the RH-23 peptide was able to reach the MLV surface, and then gradually make its way into the hydrophobic core of the phospholipid bilayers.

### 3.2. LB Measurements

**Figure 3:**
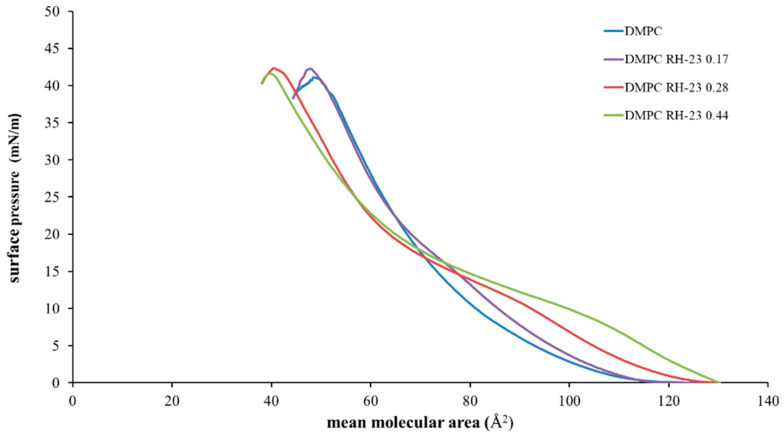
Surface pressure (mN/m)/mean area per molecule (Å^2^) isotherms of DMPC monolayers in the presence of RH-23 at different molar fractions in the subphase, at a temperature of 37 °C and pH 7.4.

The interaction between the RH-23 peptide and DMPC monolayers was studied using the LB technique and the mean molecular area/surface pressure isotherms of DMPC and DMPC/RH-23 monolayers in the subphase were recorded. In Figure 3, we show the mean molecular area/surface pressure isotherms of DMPC monolayers and DMPC monolayers in the presence of RH-23 at different molar fractions in the subphase. The DMPC showed a gas phase up to a molecular area value of approximately 110 Å^2^ and an expanded liquid phase at lower molecular area values. The collapse of the monolayer was observed to occur at approximately 40 mN/m.

The isotherms of the DMPC monolayers in the presence of RH-23 in the subphase showed various different features compared to the isotherm of the DMPC. Indeed, the isotherms moved towards higher values of the mean molecular area up to a surface pressure of approximately 18 mN/m, while for higher values of surface pressure, they moved towards lower values of molecular area. In these monolayers, a variety of states were observed, namely a gas state, an expanded liquid state, an expanded liquid/condensed liquid transition, and a condensed liquid phase. As the RH-23 molar fraction increased, an expanded liquid/condensed liquid transition was observed at higher values of the mean molecular area and at lower values of surface pressure, with respect to DMPC. This was a clear sign of the interaction of the RH-23 peptide with DMPC. For low values of surface pressure, RH-23 could insert itself in between DMPC molecules and cause an expansion of the monolayer, but when the monolayer was in a compressed state at high values of surface pressure, RH-23 could be expelled from the monolayer [46].

**Figure 4:**
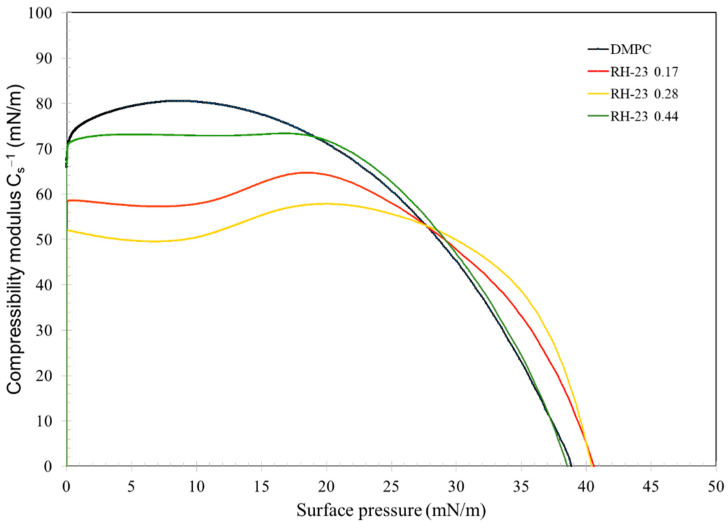
Compressibility modulus curves of DMPC monolayers with RH-23 at different molar fractions in the subphase, as a function of the surface pressure.

The compressibility modulus (Equation (1)), which gives an indication of the elastic behavior of the monolayer [47], was evaluated using the following Equation:

(1) $$C_{s}^{-1}=-A\left(\frac{\delta \pi }{\delta A}\right)$$

where *A* is the average area per molecule and π is the surface pressure. $C_{s}^{-1}$ is related to the state of the film: the larger the value, the more rigid and less compressible the monolayer. A reduction in the compressibility modulus (Figure 4) indicated that the film underwent fluidization [47,48].

RH-23 caused a decrease of the compressibility modulus of the DMPC monolayer. As a result, the DMPC monolayer was in a more fluid state in the presence of RH-23 in the subphase than in its absence.

**Figure 5:**
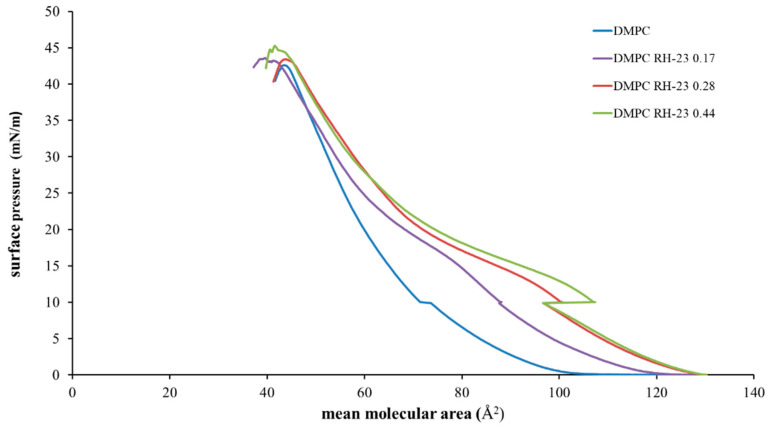
Surface pressure (mN/m)/mean molecular area (Å^2^) isotherms of DMPC monolayers in the presence of RH-23 at different molar fractions in the subphase with a pause of 30 min at 10 mN/m, at a temperature of 37 °C and pH 7.4.

**Figure 6:**
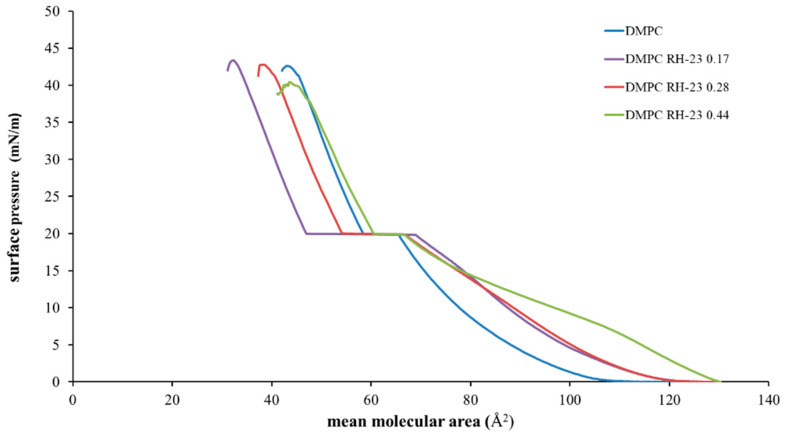
Surface pressure (mN/m)/mean molecular (Å2) isotherms of DMPC monolayers in the presence of RH-23 at different molar fractions in the subphase with a pause of 30 min at 20 mN/m, at a temperature of 37 °C and pH 7.4.

**Figure 7:**
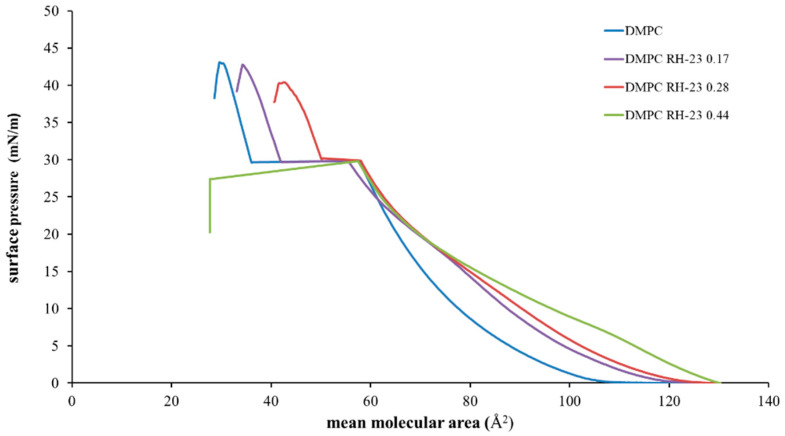
Surface pressure (mN/m)/mean molecular area (Å2) isotherms of DMPC monolayers in the presence of RH-23 at different molar fractions in the subphase with a pause of 30 min at 30 mN/m, at a temperature of 37 °C and pH 7.4.

To estimate the ability of the RH-23 peptide present in the subphase to interact with the DMPC monolayer over time, isotherms were recorded before and after maintaining the surface pressure for 30 min at 10, 20, and 30 mN/m, respectively (Figure 5, Figure 6 and Figure 7).

The isotherms recorded with a pause at 10 mN/m (Figure 5) moved towards higher values of mean molecular area with increasing peptide molar fraction. Hence, the RH-23 peptide interacted with the DMPC monolayer and affected its physicochemical properties. In the isotherms recorded with a pause at 20 mN/m (Figure 6), there was a decrease in the mean molecular area values both in the isotherm of DMPC alone and in the isotherms of DMPC in the presence of RH-23 during the pause.

In the presence of RH-23 peptide, there was a stronger decrease in the values of the average molecular area, which was inversely proportional to the molar fraction of RH-23. Hence, RH-23 interacted with the DMPC monolayer. In the isotherms recorded with a pause at 30 mN/m (Figure 7), a decrease in the average molecular area with peptide molar fraction was observed over time. The maximum decrease occurred in the absence of RH-23.

When the peptide was present, the decrease in the average molecular area recorded was less pronounced. The results obtained suggested that at the surface pressure of 10 mN/m, there was a low density of DMPC molecules in the monolayer; therefore, RH-23 could enter the monolayer and cause an expansion of the monolayer itself. Furthermore, at higher amounts of RH-23 in the subphase, more RH-23 molecules could be inserted into the monolayer causing greater expansion. At higher pressures, when the DMPC molecules were in a more compact state, RH-23 could cause the desorption of molecules from the monolayer.

**Figure 8:**
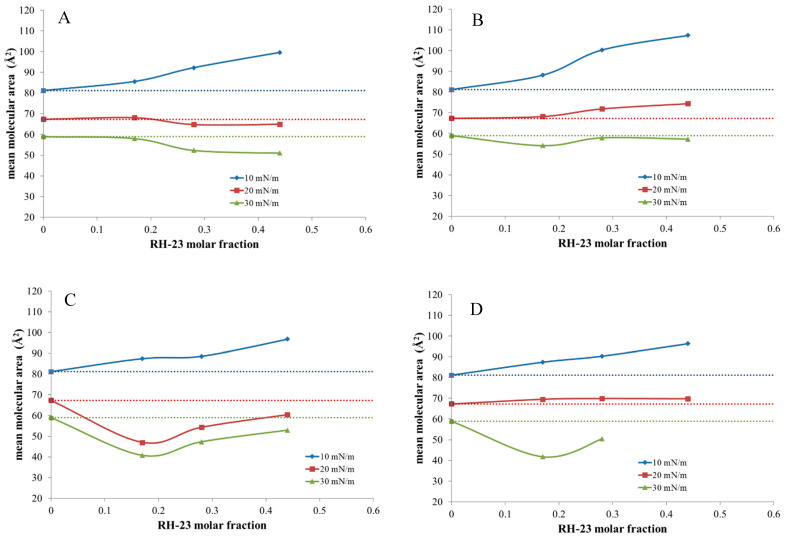
Average area per molecule (Å^2^) of DMPC monolayers with RH-23 at increasing molar fractions in the subphase, as a function of the RH-23 molar fraction, at 10, 20, and 30 mN/m surface pressure. (**A**) is without pause, (**B**) is with the pause at 10 mN/m, (**C**) is with the pause at 20 mN/m and (**D**) is with the pause at 30 mN/m.

Interesting information about the behavior of DMPC monolayers in the presence of RH-23 in the subphase was obtained by plotting the molecular area as a function of the RH-23 molar fraction at different surface pressures (Figure 8).

In the panels of Figure 8, the dashed line represented the behavior of the DMPC monolayer in the absence of RH-23. Figure 8A shows the values of the experiments carried out without pause. At 10 mN/m, RH-23 caused positive deviations and, therefore, an expansion of the monolayer at all the molar fractions explored. At 20 and 30 mN/m, RH-23 peptide at the lowest molar fraction did not seem to affect the behavior of DMPC, while at higher molar fractions, it caused a negative deviation. Figure 8B, 8C, and 8D show the values of the experiments carried out with the pause. In these panels, the molecular area values were those recorded at the beginning of the pause at the corresponding surface pressures. In the experiments performed with a pause at 10 mN/m (Figure 8B), a positive deviation was visible when RH-23 was present at all molar fractions at pressures of 10 and 20 mN/m, while at 30 mN/m, a negative deviation occurred. In the experiments performed with a pause at 20 mN/m (Figure 8C), the peptide caused a positive deviation only at 10 mN/m, while a negative deviation was observed at other surface pressures. In the experiments performed with a pause at 30 mN/m (Figure 8D), the peptide caused a positive deviation at 10 and at 20 mN/m, while it yielded a negative deviation at 30 mN/m. These results indicated that the effect of RH-23 on the DMPC monolayer varied as a function of its molar fraction and surface pressure.

**Figure 9:**
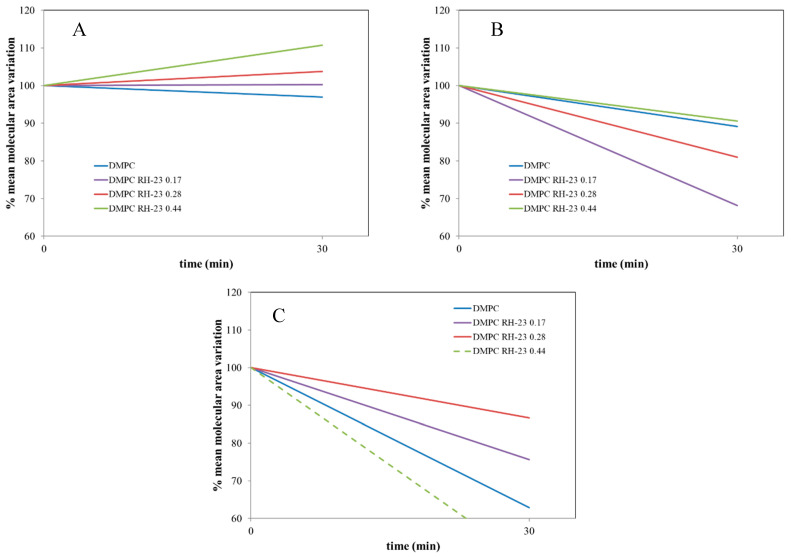
Percentage variations in the area per molecule of the DMPC monolayer in the presence of RH-23 at different molar fractions in the subphase during the 30 min pause at 10 mN/m (**A**), 20 mN/m (**B**) and 30 mN/m (**C**).

To obtain more information about the interaction between DMPC and RH-23, variations in the molecular area after an interval of 30 min were evaluated while the surface pressure was kept constant. Looking at the data with a pause at 10 mN/m (Figure 9A), we see that the molecular area slightly decreased in the DMPC monolayer without RH-23 in the subphase. This behavior indicated that DMPC molecules desorbed from the monolayer. In the presence of RH-23 in the subphase, an increase in the molecular area was observed that increased with the increasing molar fraction of RH-23. This could indicate that, since the DMPC monolayer is in a very expanded state at 10 mN/m, RH-23 gradually inserted itself into the monolayer with a consequent expansion of the monolayer itself. In the experiments carried out with a pause at 20 mN/m (Figure 9B), a different behavior was observed. In fact, RH-23 caused a reduction of the molecular area compared to the monolayer of DMPC alone. At this surface pressure, the molecules of the monolayer would be in a state to allow RH-23 expulsion in the subphase. In the experiments performed with a pause at 30 mN/m (Figure 9C), the molecular area decreased, and the decrease was greater when RH-23 was in the subphase. At this surface pressure, in the presence of highly compacted molecules, RH-23 could be expelled from the monolayer but remained anchored to DMPC molecules with a stabilizing effect.

## 4. Conclusions

In this work, the interaction of the RH-23 peptide with DMPC organized in MLV_S_ and monolayers was studied using differential scanning calorimetry and the Langmuir-Blodgett technique. Two types of experiments were performed using MLVs. The MLVs were prepared in the absence and presence of RH-23 at increasing molar fractions and the effect of RH-23 on the MLVs was evaluated by DSC. RH-23 interacted with MLVs and affected their behavior, causing a stabilization of the gel phase, which increased with increasing RH-23 molar fraction. To evaluate whether RH-23 can interact with MLV phospholipid bilayers in an aqueous environment, the possible interaction was evaluated over time using DSC. The results obtained indicated that RH-23 interacted with MLV phospholipid bilayers. Using the Langmuir-Blodgett technique, DMPC monolayers were prepared, and the ability of RH-23 present in the subphase to interact with DMPC was studied. The experiments were performed at different surface pressure values and with different RH-23 molar fractions. The results indicated that RH-23 inserted itself into the monolayer and interacted with DMPC molecules. Depending on the surface pressure and its molar fraction, RH-23 could cause a desorption of the monolayer molecules or stabilize the monolayer. The results of this investigation break ground for further experiments to deeply understand the complex mechanisms underlying the gradual interaction of the peptide at different concentrations and conditions using DMPC-based membrane models. Together, this information could be used in future biochemical studies of interplays between RH-23 and biological membranes in both physiological and pathologic conditions. The desirable endpoint would be the thorough design of RH-23-based targeting carriers for theragnostic applications.

## Figures and Tables

**Table 1:** Variations in temperature and enthalpy values (ΔH) of the transition peaks of MLVs in the absence and presence of RH-23 at increasing molar fractions.

RH-23 Molar Fraction	Temperature (°C)	∆H (Jg^−1^)
0.0	24.58	−31.59
0.03	24.83	−31.81
0.045	24.89	−32.80
0.06	24.86	−32.18
0.09	25.75	−32.42
0.12	26.58	−23.82

## Data Availability

Data were generated at the Department of Drug and Health Sciences, University of Catania. Data supporting the results of this study are available from the corresponding authors on request.
